# To Combat Industrial Fatigue

**Published:** 1920-09-25

**Authors:** 


THE PUBLIC WELL-BEING—(*continued*).

### To Combat Industrial Fatigue.

The proposal to found a school of industrial medicine in London is one that should receive the sympathetic support of the whole community. It is a fact that before the war the deeply interesting and highly important subject of industrial fatigue was never thoroughly investigated on a scientific basis in this country. But the conditions and experiences, as well as the after-effects of the war, have proved the necessity for combating the consequences of fatigue in order both to improve and at the same time economise output, and to better the lot of the working population. There is undoubtedly great scope for investigation on these lines in a school of industrial medicine. A distinguished man said not long ago that there are almost as many kinds of exhaustion as there are diseases. When we have discovered the true nature of exhaustion and found a satisfactory process of classification we shall have reached another milestone towards the prevention of disease. The project now commended to the notice of the public is in line with the new attitude of humane consideration for the worker, both mental and manual. The truth is at last sinking in that, taking material grounds alone, a man is a better worker when he is regarded as a man and not merely as a machine. The present tendency to go slow on the part of large elements of the population—to adopt what is commonly described as a "ca' canny" policy—is largely attributable to nervous reaction after the strain of a tremendous war. As soon as this effect has passed away better conditions of labour and a closer and more sympathetic relationship between master and man will begin to show results that will benefit the nation as a whole.

Incidentally it may be of practical service in the special direction of assisting the State to formulate wise and appropriate regulations for preserving the welfare of workers in factories and workshops. The State would undoubtedly be sympathetically disposed to any recommendations arising from so authoritative a source. At the present time the Home Office is showing itself to be fully alive to the importance of increasing the amenities of daily labour. The new order which it has just issued is admirable. It will not greatly affect the up-to-date and humane employer, but it will call to attention those who failed to realise their responsibilities. This order makes it obligatory to provide protective clothing for those employed in wet or dirty processes; it stipulates for the provision of mess-rooms, with adequate means for cooking and warming food and boiling water; it insists upon washing facilities for the employees, and also for the provision of seats, so that those whose work is done standing may be able to take advantage of any opportunity for resting which may occur in the course of their employment (a very important provision for women workers); and, furthermore, it makes obligatory the establishment of proper first-aid arrangements, and specifies the nature of the ambulance and other appliances which are to be kept ready for accidents.

